# Investigation of the knowledge of South African high school rugby coaches on concussion and the return-to-play protocol

**DOI:** 10.17159/2078-516X/2022/v34i1a12255

**Published:** 2022-01-01

**Authors:** NC Abel, CC Grant, DC Janse van Rensburg

**Affiliations:** 1 Section Sports Medicine, Faculty of Health Sciences, University of Pretoria, Pretoria, South Africa; 2 International Netball Federation, Manchester, UK Medical Board Member, UK

**Keywords:** youth, sports concussion, BokSmart, Maddocks questions, traumatic brain injury

## Abstract

**Background:**

Coaches are pivotal in the management of concussed players. Assessing the knowledge of high school rugby coaches with regard to concussion management will enable relevant future education on this topic to be covered.

**Objectives:**

To investigate the knowledge of South African high school rugby coaches on concussion symptom recognition, knowledge and stepwise return-to-play (RTP) protocols.

**Methods:**

A cross-sectional descriptive study involving 143 first team, high school rugby coaches was completed via an electronic questionnaire. Independent variables included coach demographics, qualifications, experience, BokSmart accreditation, and school size. Dependent variables included knowledge scores on concussion symptoms, general concussion knowledge, stepwise RTP and Maddocks questions. Relationships between total scores for different demographic groupings were established using non-parametric techniques.

**Results:**

The coaches had high general, symptom and overall concussion knowledge scores (77% – 80%) in contrast with low RTP scores (62%) and very low Maddocks questions knowledge scores (26%). The 35–44-year age group received top scores for symptom recognition (p=0.034) and total concussion knowledge (p=0.041). Larger category school coaches (p=0.008) and BokSmart accredited coaches (p=0.041) outperformed all other coaches in general concussion knowledge and total knowledge, respectively. However, respondents were not familiar with emotional symptoms or the importance of cognitive rest after a concussion. Educational programmes were the most popular knowledge source for coaches.

**Conclusion:**

In general, coaches presented with good general concussion knowledge but lesser expertise on emotional symptoms, cognitive rest and RTP management. Modifiable predictors of knowledge included the expansion of BokSmart accreditation, focussing information sessions on smaller rugby size schools and the education of coaches younger than 35 years or older than 45 years of age.

Research suggests that players are more likely to report a concussion to their coaches than to anyone else.^[1–6]^ The role of the coach is therefore central in creating a culture that supports seeking medical assistance and adherence to treatment. Studies by Mathema et al. and Mrazik et al. showed that rugby and hockey coaches scored above 80% to 90% in overall concussion knowledge, with scores in the 60% to 70% range for return-to-play (RTP) guidelines.^[1,2]^ South African amateur rugby union coaches scored 74% when their concussion knowledge was tested by van Vuuren et.al. However, this study also showed that the coaches have insufficient knowledge regarding RTP.^[6]^

Although the literature shows that coaches have a good general knowledge of concussion, they may occasionally make inappropriate decisions regarding its management. For example, international research showed that 40% of coaches are willing to put pressure on concussed players to remain playing, while 28% of coaches might pressure the medical staff to allow a concussed player to continue with the game. ^[6]^ Furthermore, 39% of coaches may allow a player to continue to play if the player indicated being fine after being knocked out.^[2]^ Coaches are often influenced by the importance of the game when making decisions to allow a concussed player to continue playing. In wrestling, 27% of coaches admitted that they would allow a potentially concussed wrestler to continue wrestling in a regional qualifier compared to 12% of coaches who stated they would allow this to happen in the first match of the season.^[7]^

Studies covering the testing of the knowledge of players and coaches reported certain collective misconceptions and gaps in their knowledge. Players often do not know that sleep disturbance and emotional symptoms are related to concussions.^[8,9,10]^ The importance of cognitive rest is often not well understood as part of the recovery process,^[1,4]^ and there are also misunderstandings about the significance of a player’s loss of consciousness during a game. In studies by Mathema et al. ^[1]^ and Guilmette et al.,^[11]^ only 55% of players and 61% of coaches respectively, felt that a player should be removed from the field after being knocked unconscious.

Little is known about this topic in rugby union games at school level, where the coaches are often teachers and not professional coaches. The BokSmart National Rugby Safety Programme - a joint rugby safety initiative between SA Rugby and the Chris Burger/Petro Jackson Players’ Fund – was founded in South Africa in 2009. An original development priority of the BokSmart Programme was to improve player safety in specific rural, as well as urban, school settings by increasing the knowledge of rugby coaches, referees and players. Coaches are required to keep their BokSmart accreditation up to date. Research on the subject in the South African high school rugby setting is sparse. This study was designed to investigate the knowledge of South African high school rugby coaches on concussion symptom recognition, general concussion knowledge and stepwise RTP protocols.

## Methods

### Study design

A cross-sectional, descriptive study was conducted after approval was granted by the institutional research ethics committee of the Faculty of Health Sciences, University of Pretoria. An invitation to participate in the project was addressed to high school coaches representing all South African provinces. The letter, including the study background, confirmation of institutional ethical approval, consent form, questionnaire, and letter of support from the South African Rugby Union (SARU) medical committee and the Director of Sport Concussion South Africa, was distributed on five separate occasions (four to six weeks apart) during an eight-month period. Data from the submitted questionnaires were exported to a Microsoft Excel file and analysed. The anonymity of all respondents was maintained by including only a study number for participants and no personal details. The questionnaire was piloted prior the start of the study, using eight coaches at local high schools. Coaches recorded their questionnaire completion times and provided comments on the questionnaire’s contents and usability.

### Data

Independent variables (predictors) included the demographics of the coaches, coaching qualifications, years of experience as a coach, BokSmart accreditation status, and the size of the school where the coach coached. Schools were categorised as ‘small’ if they had less than five rugby teams; ‘medium’ if between five and ten teams were active; and ‘large’ if more than ten teams were present. Dependent variables (outcomes) that were measured included the scores on the knowledge of concussion symptoms, general concussion knowledge, knowledge of the stepwise RTP, Maddocks questions, and knowledge sources from which the coaches obtained their knowledge. Symptom recognition, general concussion knowledge questions and sources of knowledge were assessed from validated questionnaires.^[2,5,9,11]^ Emotional symptoms were specifically included in the list of symptoms to investigate the understanding of these symptoms in relation to concussion.^[4]^ RTP questions were designed by the researcher, together with open-ended questions, on the coach’s knowledge of the Maddocks questions. The questionnaire is attached as Supplement A.

### Statistical analysis

Knowledge scores were calculated as a combination of the seventeen Yes/No questions on symptom recognition and eight True/False questions on general questions about the coaches’ attitude towards concussion. The maximum knowledge score was therefore 25 points.

True/False questions were used to obtain RTP scores and included scenarios regarding same-day RTP (four questions), time out from sport following concussion (four questions), symptom occurrence during stepwise RTP (four questions), cognitive rest (four questions) and the importance of seeing a doctor (five questions). The maximum RTP score was 21 points. The knowledge of the Maddocks questions was tested through open-ended questions and scored a maximum of five points.

Frequency tables, including counts and percentages, were used to present the responses to the incorporated demographic questions. Means and standard deviations (SDs) were calculated for the total scores of each subsection. Due to the presence of small and/or uneven sample sizes within the groups, relationships between total scores for different demographic groupings were also established using non-parametric statistical techniques. When two groups were compared, the Wilcoxon two-sample test was used, and when more than two groups were compared, the Kruskal-Wallis test was used, followed by a Bonferroni multiple comparison test to identify which pairs differed significantly.

## Results

### Coaches’ background information

A total of 640 surveys were distributed and 143 responses were returned. The coaches’ number of years of experience is presented in Table 1. Most of the representation (47%) came from three rugby unions, including the Golden Lions (17%), Boland (16%) and the Blue Bulls (14%).

### Coach age, level of education and accreditation

Table 2 shows that more than half of the coaches (96 of 143) completed Level 2 or Level 3 coaching courses; however, seven percent of coaches (10 of 143) had completed no coaching courses. Most of the 35–44-year-old coaches (15 of 37) with more years of experience are from the large category schools (41%). The small category schools have a larger number of the coaches (6 of 13) under 24-years-of- age (46%). Most (95%) of the coaches are BokSmart accredited (136 of 143), but only 62% of the <24-year age group reported BokSmart accreditation (8 of 13) and none of the below 34-years old progressed to the third level of the World Rugby course.

### Concussion symptom recognition

Figure 1 shows the symptoms identified by coaches as the most common signs and symptoms of concussion, and the percentages of coaches who were able to recognise them (ranked from most to least).

Headache, confusion, loss of consciousness and dizziness were recognised as symptoms and signs of concussion by more than 90% of the respondents. Amnesia was recognised by 73% of respondents, while sleep disturbance was only recognised by 55% of participants. Emotional symptoms such as irritability (41%) and sadness, including inappropriate crying, were less likely to be recognised as concussion symptoms (24%).

From the open-ended questions on the two most common symptoms of concussion, the most recognised symptom was confusion (64%). This was followed by dizziness and nausea (35%) and headaches (32%). Amnesia (14%) and loss of consciousness (8%) were not rated as a common symptom or sign of concussion. Emotional symptoms were mentioned only twice and sleep disturbances were not mentioned at all as common symptoms of concussion.

### General concussion knowledge

More than 90% of the coaches reported that it was easier to sustain another concussion following a previous concussion. Furthermore, 95% reported that concussion is a potentially serious injury that can lead to permanent damage, that loss of consciousness was not a prerequisite for concussion to be present, and that a concussed person must see a medical doctor after the injury. Nearly 70% of the participants reported that symptoms can take time to appear after the injury. However, less than 50% reported that it was difficult to diagnose concussion and that one does not have to hit one’s head to sustain a concussion.

Table 3 shows that the average score for symptom recognition/knowledge and general concussion knowledge was very high (above 78%). However, the RTP knowledge was mediocre (62%) and knowledge of the Maddocks score was poor (26%).

### Source of knowledge

Figure 2 indicates that the most popular choice of knowledge source was educational programmes, such as the BokSmart programme (listed as 40% of first choices and 28% of second choices). The second most popular choice was knowledge gained through working with healthcare providers (39% as first choice and 24% as second choice). These two choices accounted for 68% of the first choice votes and 63% of the second choice votes. Use of the internet was a less likely used source of knowledge (45%).

### Influence of independent variables on dependant variables

Table 4 provides a concise summary of the statistically significant findings on the influence of independent variables on dependent variables. Results indicated that coaches in the 35–44-year age group, those from large category schools and those with BokSmart accreditation showed significantly higher scores regarding concussion knowledge.

## Discussion

This study is one of the first to test the concussion knowledge of South African high school rugby coaches. Participants in this study showed high general symptom recognition and overall concussion knowledge (77% to 80%). The score of 78% for symptom knowledge in this group is comparable with the scores of Welsh elite and semi-professional rugby union coaches (77%),^[1]^ and comparable with the knowledge of South African amateur club coaches (74%).^[6]^ Impressively, the score is only seven percent less than the average scores of medical professionals in the latest Canadian research.^[10]^ However, low RTP (62%) and very low Maddocks question knowledge scores (26%) were found, perhaps indicating that coaches do not have sufficient knowledge to adequately manage the RTP of concussed players. This supports the findings of van Vuuren et.al.^(6)^ in South African amateur club coaches.

BokSmart accreditation, the size of the school and the age group of the coaches were identified as predictors for superior knowledge, while coaches at small schools were identified as a possible group with sub-standard knowledge of concussion. Misunderstandings about key concepts of concussion are also true for the latter group.

### Experience and qualifications

Literature indicates that years of experience as a coach ^[2,9,11]^ and education in concussion are predictors of superior knowledge.^[4,9,11]^ The respondents in our study were experienced in years of coaching, level of coaching qualification, and BokSmart accreditation. There was also a relatively equal representation of the different sizes of schools between the coaches, making for better comparison. The large schools showed the highest percentage (39%) of their coaches between 35–44-years of age, while the small schools have the highest percentage of teachers under 24-years of age (42%). This under 24-year group also represented the highest percentage of coaches without BokSmart accreditation (62%) and is the group of coaches with the least qualifications.

### Symptom recognition and concussion knowledge scores

It is important to note that similar to international literature, the South African coaches were not aware that emotional symptoms, for example, sadness and irritability, were important symptoms of concussion.^[4,8,10]^ Recent research highlights the importance and role of emotional symptoms and the psychological impact that concussion can have on an individual.^[8]^ It is of the utmost importance to realise the significance of these symptoms during the management of concussion and the RTP phase to guard against potential fatal complications of second impact syndrome.

The high scores obtained on symptom knowledge (78%) may be attributed to the effectiveness of the ‘recognise and remove’ approach of the BokSmart programme. Athletic coaches in Alabama, United States, scored 87%,^[4]^ and female ice-hockey team coaches in Canada scored 88% on similar assessments.^[8]^ Regarding the Canadian results that tested the knowledge of all role players, coaches scored 81%, only slightly lower than the 85% scored by medical professionals in the same study.^[10]^ The excellent Canadian results are testament to the legislated concussion education for high school sports coaches in Canada.

The general concussion knowledge score of 80% in our study compares well to the scoring in this category in the literature, ^[8,9]^ indicating that the tested coaches have a sound knowledge of the recognition of concussion. Coaches are aware that the player does not have to lose consciousness to sustain a concussion and they are aware that the player must see a doctor following a concussive incident. However, our group performed poorly when compared to two other studies that asked the same questions ^[8,2]^. Only half of the coaches understood that hitting your head was not essential to sustaining a concussion and only 70% knew that concussion symptoms can develop over time. In the literature, most of the respondents from the Canadian study answered these two statements correctly.^[2,8]^

Researchers in recent years have started more extensive investigations around RTP knowledge of coaches.^[1,6,8,10]^ Previous research investigated RTP knowledge with only a few statements on the category.^9^ In the literature on rugby union, coaches typically scored worse on their RTP knowledge than in other categories,^[1,6]^ which aligns to the results of the current investigation where coaches only scored 62% on average in the RTP category. Welsh elite and semi-professional coaches scored 71% by comparison,^[1]^ It can be expected that a 62% average result is not indicative of adequate knowledge to make sound decisions on RTP.

By comparison, RTP knowledge tested in the same manner in the Canadian study revealed an average score of 91%.^[10]^ This score was not only for the coaches as a group, but the average score for all the role players in the management of concussions. It therefore seems possible to improve coaches’ knowledge on RTP to a higher level and indicates an opportunity to educate South African high school coaches in this regard. As in Canada, the solution might lie in legislated concussion education for sports coaches in South Africa.

Even though the coach as a role player does not make the final decision on RTP, his/her knowledge must be sufficient to ensure player safety. Knowledge around concussion RTP by medical professionals might not be up to standard,^[12,13]^ and a coach needs good knowledge on the matter to be comfortable questioning decisions by medical professionals who may return a player to play or training too early. Coaches also need to monitor players’ performance after RTP and thereby the lack of knowledge on the value and implementation of cognitive rest during RTP is another significant area of concern that concurs with recommendations from other international studies.^[1,4]^

An average score of 66% for RTP is slightly lower than the scores presented in other research studies. Previous studies in rugby union indicated an assessment score of 75% for elite and semi-professional coaches.^[1]^ A total concussion knowledge score of 81%, which includes all three categories, was achieved in the research from Canada.^[10]^ The total general concussion scores are difficult to compare with most of the other research, because most of the previous research does not include RTP testing, but only symptom and knowledge testing.^[8.4]^

### Maddocks questions

Little is reported in the literature about coach knowledge of the Maddocks questions. These are specific questions that should be asked to the player on or next to the field to test their short-term memory and awareness following a potential concussive injury. These questions should be easy to remember, specific, and part of the examination of a concussed player as recommended by the Zurich Consensus Statement in 2012 and the Berlin Consensus Statement in 2016. The group average for the Maddocks questions was very low at 26% (1.3/5.0). Because of the simplicity of the questions, they should be easy to teach and remember, but it is important to know that the Maddocks questions are only a small part of the examination of the concussed player. Knowing the answers to the questions does not exclude concussion, but the opposite is more important. If the players do not know the answers, one can suspect that they are concussed and should be removed from the field.

### Preferred sources from which coaches obtain their knowledge

The participants’ preferred choices for gaining knowledge about concussion are similar to those described in other studies.^[1,8,11]^ Educational programmes and education by healthcare professionals together cover 68% of the first-choice and 63% of the second-choice votes in our study. The third most popular choice was use of the internet. In previous studies, most of the coaches felt that the best source of knowledge was that of the medical professionals with whom they work.^[1,8,11]^ Some coaches also thought that a training “kit” is helpful, while some preferred online teaching.^[8,11]^

### Independent variables

#### Age

The coaches in the 35–44-year group, significantly outscored the other groups in most of the knowledge categories, including symptom recognition (p=0.034), total knowledge, including Maddocks questions (p=0.041). This age group also represents the biggest percentage of coaches at large schools. Most of the current literature supports the fact that coaching experience ^[2,11]^ and education ^[4,9,11]^ are better predictors of knowledge as opposed to only the age of the coach.

There was no significant difference in scoring between the coaches according to their qualification levels as a coach and their years in coaching. In both categories there was a trend toward higher scores with experience and higher levels of qualification however, it did not prove to be statistically significant. Although not significant, this association does support the notion of coaching experience as a predictor of superior concussion knowledge. ^[2,9,11]^

#### Size of school

Race, socioeconomic circumstances, cost of consultations, household income and geographic location are all predictors of superior knowledge. ^[10,14]^ Elite high school athletes seem to have better overall concussion knowledge compared to athletes from lower income areas according to studies from America.^[14]^ Concurringly, private schools in South Africa report more concussions, which may be due to better knowledge and awareness of the risks involved in youth concussions.^[15]^

In South Africa, the macro/large schools are typically the schools that can afford to hire professional coaches who are not simply teachers who also coach, and they often have athletic trainers, physiotherapists, and team doctors. The smaller schools on the other hand, are often underprivileged schools with teachers that have to teach, coach, mentor and diagnose concussion. According to our study, the small schools also end up with the non-accredited, youngest, and most inexperienced coaches who scored significantly lower than coaches from larger schools on concussion knowledge in general (p=0.008). That makes this young group of coaches susceptible to unintended mismanagement of concussed players, which may result in career-ending or even fatal injuries.

The difference in general concussion knowledge scores being prevalent only in the larger category schools in this study is a reason for concern as it is the hybrid coaches at smaller schools who require better concussion knowledge and related skills.

#### BokSmart

As can be expected, concussion education is a significant predictor of superior knowledge. ^[2,4,9]^ In South Africa, concussion education for coaches is provided through the BokSmart programme and it is a regulatory requirement of the SARU that all coaches are BokSmart certified. Our results support the notion that education via this educational programme leads to superior knowledge. Although the non-accredited coaches were a small group (n=8), the results confirmed that there is a statistically significant difference in total concussion knowledge between the accredited and non-accredited coaches (p=0.041). Education and legislation through the existing educational channels, such as the BokSmart programme in South-Africa, can greatly aid in the knowledge and exposure of younger, inexperienced coaches.

### Study limitations and recommendations

As with much questionnaire-based research, the response rate was relatively low, with only 143 responses collected from a possible 640. These results can be viewed as a best-case scenario because the responders were also likely to be the coaches with better concussion knowledge and interests in the subject. A factor not investigated in our study is the role of previous personal experience with concussion as a predictor of superior knowledge, particularly since it stands to reason that previous experience with concussions may lead to superior knowledge. Although the questionnaire was tested among a small group of coaches from a rugby-playing school, minor layout errors in questionnaire format might also impact the type and quality of answers received by respondents, especially regarding the Maddock questions.

## Conclusion

This present research undertaking has shown that the participants (high school rugby coaches in South Africa) have high general, symptom-specific, and overall concussion knowledge that is comparable to other similar international studies. However, coaches’ knowledge of emotional symptoms, cognitive rest and RTP strategies/management, need to be improved and updated. The age of the coach (mostly in terms of experience), the size of the school and their BokSmart accreditation status were significant predictors of superior knowledge related to concussion. Smaller category schools, where resources are more limited, have younger coaches that show far lower percentages of BokSmart accreditation (required by SA Rugby Union Regulations), lower qualification levels and higher numbers of inexperienced coaches, putting them at a higher risk for unintentional concussion mismanagement and legal repercussions in the event of catastrophic/fatal injuries. Future education should aim to address the knowledge gaps specifically regarding cognitive rest, emotional signs and symptoms of concussion and return-to-play management. This can be achieved through an existing educational programme such as BokSmart and engagements with healthcare professionals. Legislated concussion education for all high school stakeholders, which is the case in Canada and the United States of America, can be a possible solution.

## Supplementary Information



## Figures and Tables

**Fig. 1:**
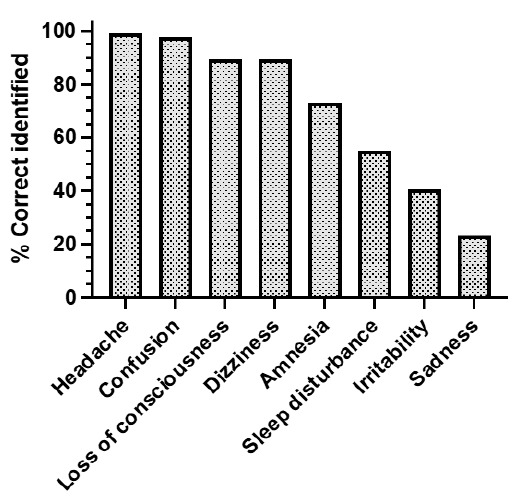
Concussion symptom or signs recognition by coaches.

**Fig. 1:**
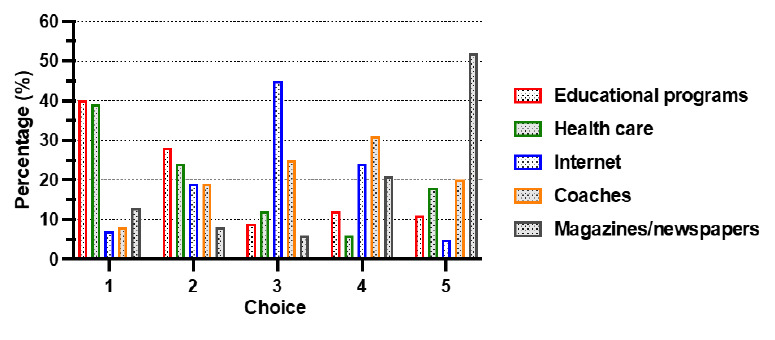
Sources of knowledge

**Table 1:** Distribution of coaches’ years of experience (n=143)

Years’ experience (%)			
< 3 years	3 – 5 years	5 – 10 years	> 10 years
5	15	19	61

**Table 2:** Age groups, level of coaching attained, school categories and BokSmart accreditation of coaches

Age (Years)	n	World Rugby Course Level				Category of School			BokSmart accreditation
		None	1	2	3	Small	Medium	Large	
< 24	13	30.8 (4)	38.5 (5)	30.7 (4)	-	46.2 (6)	23.0 (3)	30.8 (4)	61.5 (8)
25 – 34	54	7.4 (4)	31.5 (17)	61.1 (33)	-	40.7 (22)	38.9 (21)	20.4 (11)	100.0 (54)
35 – 44	37	2.7 (1)	27.0 (10)	62.2 (23)	8.1 (3)	24.3 (9)	35.1 (13)	40.6 (15)	100.0 (37)
45 – 54	30	3.3 (1)	16.7 (5)	76.7 (23)	3.3 (1)	16.7 (5)	53.3 (16)	30.0 (9)	93.3 (28)
55 – 64	9	-	-	66.7 (6)	33.3 (3)	33.3 (3)	22.2 (2)	44.5 (4)	100.0 (9)
All	143	7.0 (10)	25.9 (37)	62.2 (89)	4.9 (7)	31.5 (45)	38.5 (55)	30.0 (43)	95.1 (136)

Data are expressed as percentage % (sample number, n)

**Table 3:** Mean participant scores and percentages on various concussion knowledge questions

Variable	Maximum score	Mean (SD)	Mean score (%)	Range
Symptom recognition/knowledge*	25	19.5 (2.0)	78	13 – 24
General concussion knowledge	8	6.4 (1.1)	80	4 – 8
Return-to-play†	21	13.1 (1.5)	62	9 – 16
Maddocks score**	5	1.3 (1.2)	26	0 – 5

* Refers to Questions 8 and 10 (maximum score = 25) - Scores for symptom and general concussion knowledge.

** Refers to Maddocks score (maximum score = 5) - Scores for knowledge on Maddocks questions.

† Refers to Questions 11 – 15 (maximum score = 21) - Scores for Return-to-Play knowledge.

**Table 4:** Summary of statistical significant findings on the influence of independent variables on dependant variables

Independent (Predictor) variable	Dependent (Outcome) variable	P-value	Interpretation
Coach age group	Symptom recognition knowledge	0.034	Differences in the knowledge of age groups are significant. The 35–44-year age group scored best in all these categories
	Total knowledge score	0.041	

Size of the school	General concussion knowledge	0.0084	Large category school coaches have statistically a better general concussion knowledge than small category school coaches

BokSmart accreditation	Total knowledge score	0.041	BokSmart accredited coaches have superior total knowledge of concussion
