# Supplementary material for: Investigation of the knowledge of South African high school rugby coaches on concussion and the return-to-play protocol
Source: S Afr J Sports Med. 2022 Jan 1;34(1):v34i1a12255. doi: 10.17159/2078-516X/2022/v34i1a12255 (PMC9924564; doi:10.17159/2078-516X/2022/v34i1a12255)
Supplement: Supplementary file 1 [file 2078-516X-34-v34i1a12255-s001.pdf]

Investigation of the knowledge of South African high school rugby coaches on concussion and the return-to-play protocol

Supplement A: Concussion Questionnaire

Thank you for taking the time to complete this questionnaire, it is completely anonymous.

Participant information

1. Age:

2. Gender:

3. Completed IRB coaching course:

Level 1 – introducing rugby	
Level 2 – Developing rugby skills	
Level 3 – Analysis & preparation for coaches	
Coaching sevens	
None	

4. In which rugby union do you coach rugby? Tick the correct box.

Blue Bulls	
Boland Kavaliers	
Border Bulldogs	
Eastern Province Kings	
Free State Cheetahs	
Golden lions	
Griffons	
Griquas	
Leopards	
Pumas	
KZN Sharks	
SWD Eagles	
Valke	
Western Province	

5. How many years have you been a coach? Tick the correct box.

Less than 3 years	
3 to 5 years	
5 to 10 years	
More than 10 years	

6. In which category does your school compete? Tick the correct box.

Small school – less than 5 teams	
Medium school – between 5 and 10 teams	
Large school – more than 10 teams	

7. Do you have BokSmart accreditation? Tick the box.

Yes	
No	

8. Which of the following are symptoms of concussion?

	Yes	No
Black eye		
Abnormal sense of smell		
Amnesia		
Headache		
Abnormal taste sensation		
Tingling in the neck and shoulders		
Sharp burning neck pain		
Sleep disturbance		
Weakness in the neck		
Nosebleed		
Nausea		
Loss of consciousness		
Hyperventilating		
Dizziness		
Inappropriate crying		
Irritability		
Confusion		

9. In your experience, what are the two most common symptoms of concussion?

A ____

B ____

Please answer the next set of questions, by choosing whether each statement is true or false

	True	False
A person who has recovered from concussion is less able to withstand a second blow to the head		
It is easy to tell whether a person is concussed by the way he looks or acts		
Concussion is generally harmless and does not result in any permanent damage		
A person has to lose consciousness to sustain concussion		
A player does not have to hit his head to sustain concussion		
Symptoms of concussion will appear immediately after the injury		
Concussion is not as serious as some other injuries		
It is absolutely essential to see a doctor after concussion, even a minor concussion		

11. Your star player sustains a minor concussion and he looks ok on the field, you...

	True	False
Allow him to continue playing because he is still making the right decisions on the field		
You are not that worried because he did not lose consciousness		
The doctor is happy after a concussion time-out and you send him back onto the field		
You send him back after halftime because the game is too close to play without him		

12. A player can start a game after concussion when...

	True	False
Symptoms of last week's game cleared up within 15 minutes		
3 weeks rest after concussion		
Doing contact drills without any symptoms		
13 days out and participating in full contact practice without symptoms		

13. A player has rested for 4 weeks after concussion and has a headache after the first contact practice since his return to training, you...

	True	False
Evaluate in 24 hours and if he is OK he can play the next day		
Send to a doctor and if he is cleared he can play the next day		
If he has no complaints at the next day's practice he can play the day after		
Sit him out for another week and if he is OK at the contact practice then he can play		

14. A player has confirmed concussion on the last game before the examinations. Your advice to him after the game would be...

	True	False
Keep his phone on during the weekend because you want to text him to find out if he is doing well		
Do not study and go for a walk the next day to see how he feels after some exercise		
Do not study, rather relax and watch some television		
Try to study for the Monday's examination, and let the school know if it is too difficult		

15. A player has suffered concussion, but feels good after the game.

	True	False
Can go home, rest and take fluids and see a doctor if he gets worse		
Start next weeks' game because he did well at practice		
You allow him to play in the final provincial trials 4 days after the injury because his father and the family doctor agrees that he is OK		
You are not worried about him because he looks fine and, after all, did not even lose consciousness		
Because his irritating mother will bug you forever, you tell her if she really wants to take him to a doctor for a minor concussion, she must go ahead		

16. What are the Maddock questions?

____

____

17. From which of the following sources are you most likely to obtain your knowledge on concussion. List from most likely to least likely from one to five (1 = least likely to 5 = most likely).

Magazines/Newspapers	☐
Other coaches	☐
Internet	☐
Rugby educational programs	☐
Healthcare workers/Doctors	☐
